# 2181: Patient preferences and attitudes regarding next-generation sequencing results: scoping review of the literature

**DOI:** 10.1017/cts.2017.250

**Published:** 2018-05-10

**Authors:** Matthew Neu, Sara Knight

**Affiliations:** University of Alabama at Birmingham, Birmingham, AL, USA

## Abstract

OBJECTIVES/SPECIFIC AIMS: Although the clinical utility of whole genome sequencing (WGS) is increasing, a gap exists between what WGS can deliver in quantity of genomic information and what results can be interpreted that patients and community members would find meaningful. Given the potential for incidental findings and variants of uncertain significance, an emphasis should be placed on understanding patient preferences towards receiving WGS results. To identify the current knowledge base on WGS preferences, we performed a scoping review. METHODS/STUDY POPULATION: A search on PubMed using terms “WES,” “WGS,” “genome sequencing,” ”attitudes,” and “preferences” identified survey research between 2012 and 2016. Summaries of population, sample, variables, and results were tabulated. RESULTS/ANTICIPATED RESULTS: Of 13 studies identified, 6 surveyed community members, 6 included medical professionals, and 2 surveyed cohorts with a specific medical condition. Only 1 study used a nationally representative sample and no study focused on a medically underserved population. Patients were most interested in receiving medically actionable results, yet preferred to have access to all available data if desired. Genetics professionals are more conservative with the return of incidental and uncertain findings. DISCUSSION/SIGNIFICANCE OF IMPACT: Existing surveys have limited representation of the US public. Future studies focused on medically underserved populations would provide a deeper understanding of attitudes and preferences toward WGS.

